# Comparison of upper gastrointestinal fluoroscopy versus computed tomography for evaluation of post-operative leak in a bariatric surgery patient

**DOI:** 10.1259/bjrcr.20160076

**Published:** 2016-07-22

**Authors:** Tim Xu, Nicholas Rosculet, Kimberley Steele, Martin Auster

**Affiliations:** ^1^Johns Hopkins School of Medicine, Baltimore, MD, USA; ^2^Department of Surgery, Johns Hopkins Bayview Medical Center, Baltimore, MD, USA; ^3^Department of Radiology, Johns Hopkins Bayview Medical Center, Baltimore, MD, USA

## Abstract

Bariatric surgery patients commonly undergo post-operative fluoroscopic evaluation for complications, including leaks, in order to progress with oral intake and recovery. As one of the most severe and potentially life-threatening complications, leaks occur in as many as 5% of bariatric surgery patients. Several characteristics of these patients complicate the detection of leaks, including large body habitus and limited mobility. The early detection of leaks can lead to significant reductions in morbidity and mortality in bariatric surgery patients. In a retrospective case series of 619 patients, of whom 20 had experienced a leak, CT scan had a sensitivity of 95% and specificity of 100%, while upper gastrointestinal (UGI) evaluation had an inferior sensitivity of 79% and specificity of 95%. In addition to greater sensitivity and specificity, CT scan can identify other complications, such as abscesses and bowel obstructions. Also, UGI evaluation is notably more dependent on patient and technologist compliance, resulting in suboptimal examinations. UGI, on the other hand, may help further define the size and more precise location of the leak, but typically cannot be performed until the following day if the patient becomes symptomatic at night. We propose that CT evaluation, used in combination with UGI, may increase the overall sensitivity of detecting a leak, thereby improving patient outcomes and decreasing hospital utilization.

## Background

Upper gastrointestinal (UGI) fluoroscopic evaluation in a bariatric surgery patient is commonly used to evaluate for post-operative complications such as an anastomotic leak and/or leak from a staple line. Although laparoscopic sleeve gastrectomy is a relatively safe bariatric procedure, post-operative leak is one of the most severe and potentially life-threatening complications occurring in an estimated 0.4–5.3% of all bariatric surgery patients.^1–3^ Leaks most commonly occur near the gastro-oesophageal junction and typically present in patients in the perioperative period and after initial discharge from the hospital. A universally accepted clinical algorithm for diagnosis remains to be developed, and clinical suspicion remains important for tests. From a radiographic standpoint, there are several key characteristics that make detection of leaks more difficult in patients who are undergoing bariatric surgery. First, because of a patient’s large body habitus, X-ray penetration is suboptimal. Second, positioning on the fluoroscopic examination table is often suboptimal because patients have post-operative pain with resultant limited mobility. The patient’s mobility may also be affected by the sedative effects of pain medication.

We present a case in which UGI fluoroscopy evaluation was carried out as the first diagnostic imaging test to rule out leak from a staple line in a patient who was 12 days out from revisional bariatric surgery (conversion from laparoscopic adjustable gastric band to laparoscopic vertical sleeve gastrectomy). The UGI evaluation provided inconclusive results, which led to the patient undergoing a CT scan of the abdomen and pelvis with oral non-ionic contrast. We propose that for these fresh post-operative bariatric surgery patients, fluoroscopy may not always be diagnostic and that UGI in combination with CT should be considered in the diagnostic algorithm for ruling out a leak.

## Case presentation

A 34-year-old Caucasian female, who had originally undergone an uneventful laparoscopic adjustable gastric band 4 years ago, presented to the bariatric surgery clinic with inability to tolerate solids. A work-up revealed that the laparoscopic band remained in good position but the patient had oesophagitis and gastritis, causing swelling of the mucosa at the band site. The fluid was removed from the reservoir, and the patient was treated conservatively with anti-reflux medication and a full liquid diet. After 2 weeks of treatment the patient’s symptoms improved. After careful consideration, she wished to undergo revisional surgery converting the laparoscopic adjustable gastric band to a laparoscopic vertical sleeve gastrectomy. The patient moved through the appropriate multidisciplinary team approach and was found to be an appropriate candidate for surgery. She underwent laparoscopic removal of the adjustable gastric band and conversion to a laparoscopic vertical sleeve gastrectomy without complications. Her post-operative course was uncomplicated and she was discharged on post-operative day 3.

**Figure 1.**
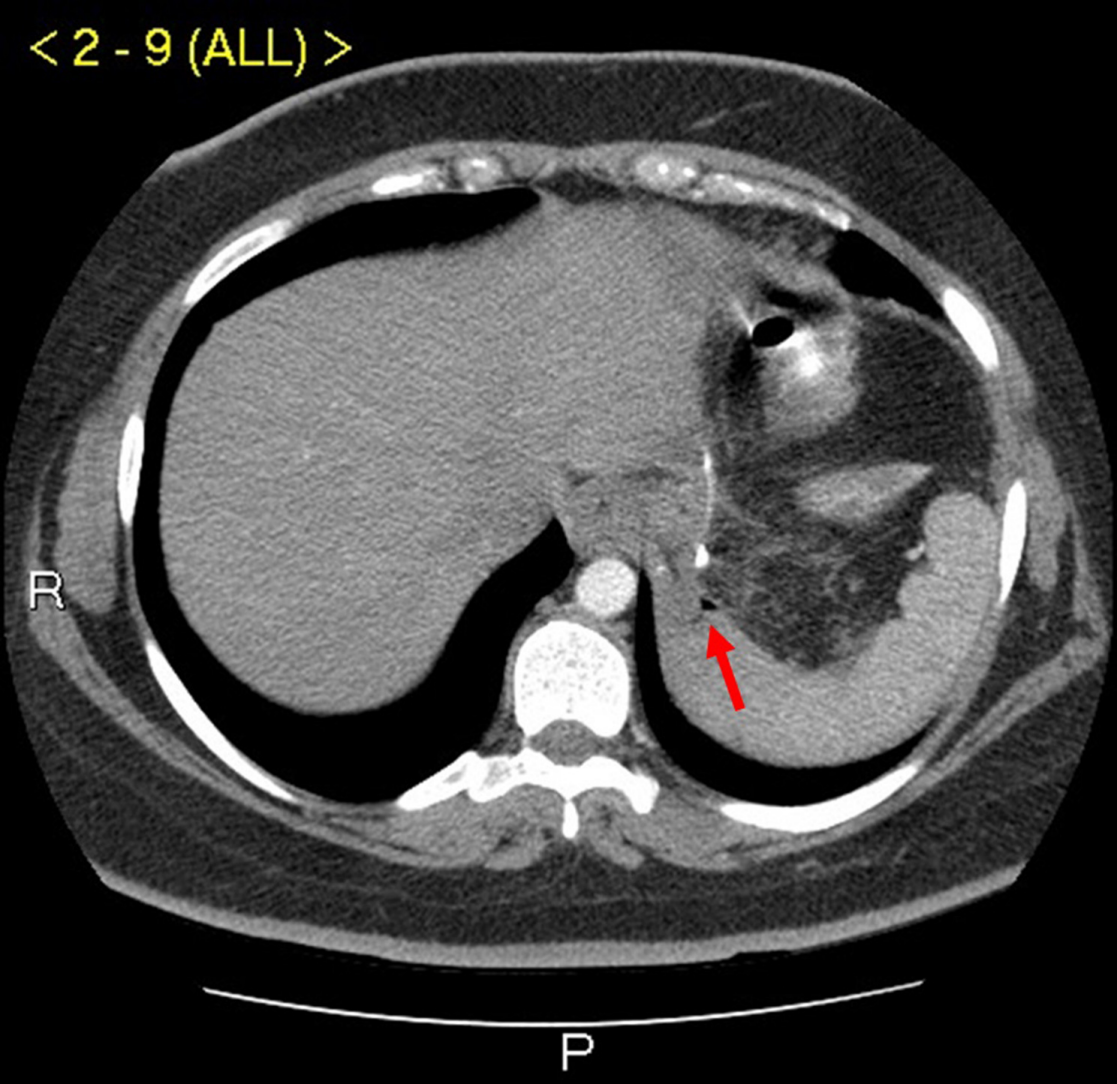
CT scan with intravenous and oral contrast showing air and a faint suggestion of oral contrast outside of the suture, late phase (arrow). P, posterior; R, right.

**Figure 2.**
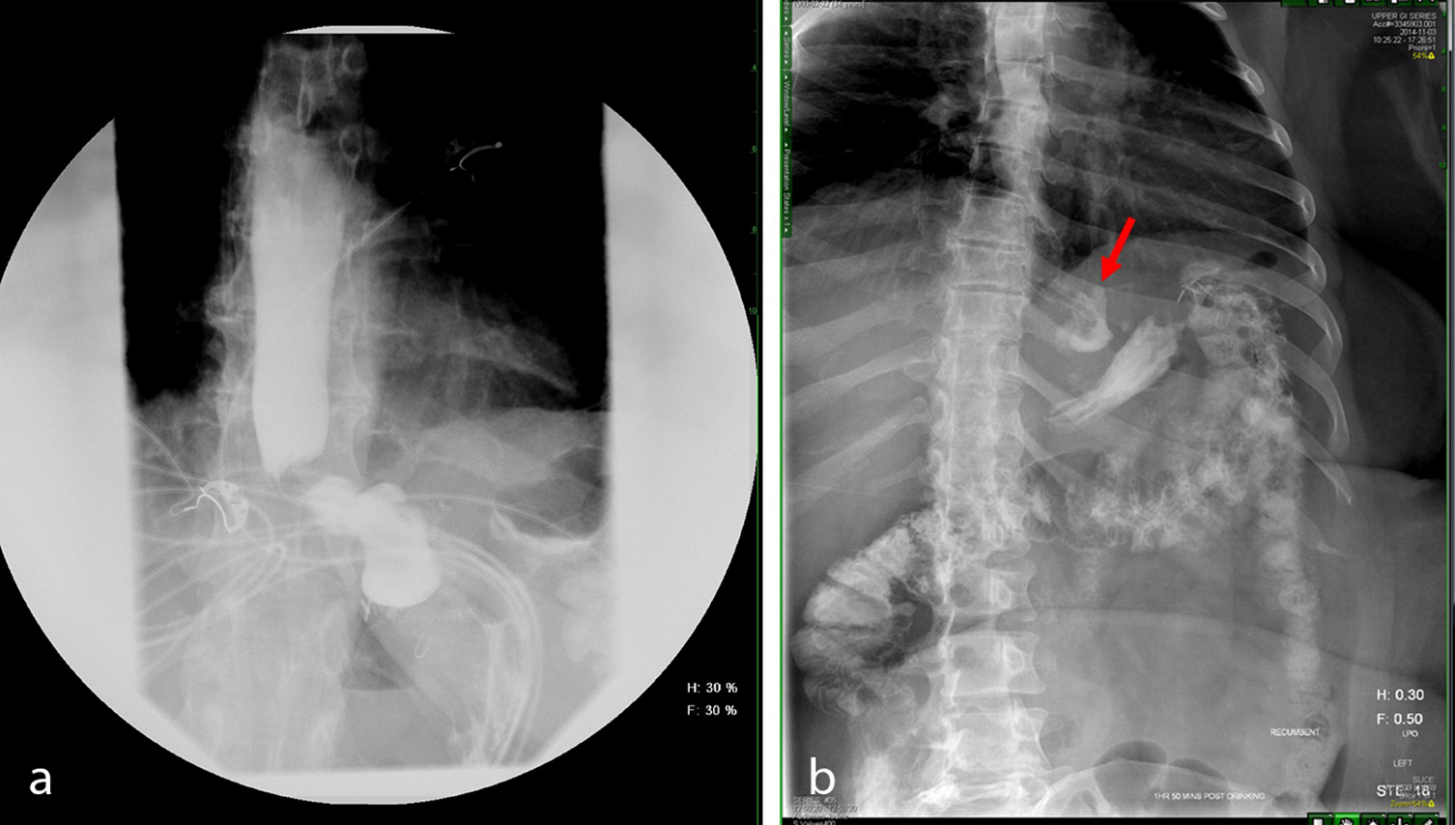
(a) Upper gastrointestinal series, early phase. (b) Upper gastrointestinal series, late phase. Arrow indicates extravasated extraluminal contrast representing leak on CT.

**Figure 3.**
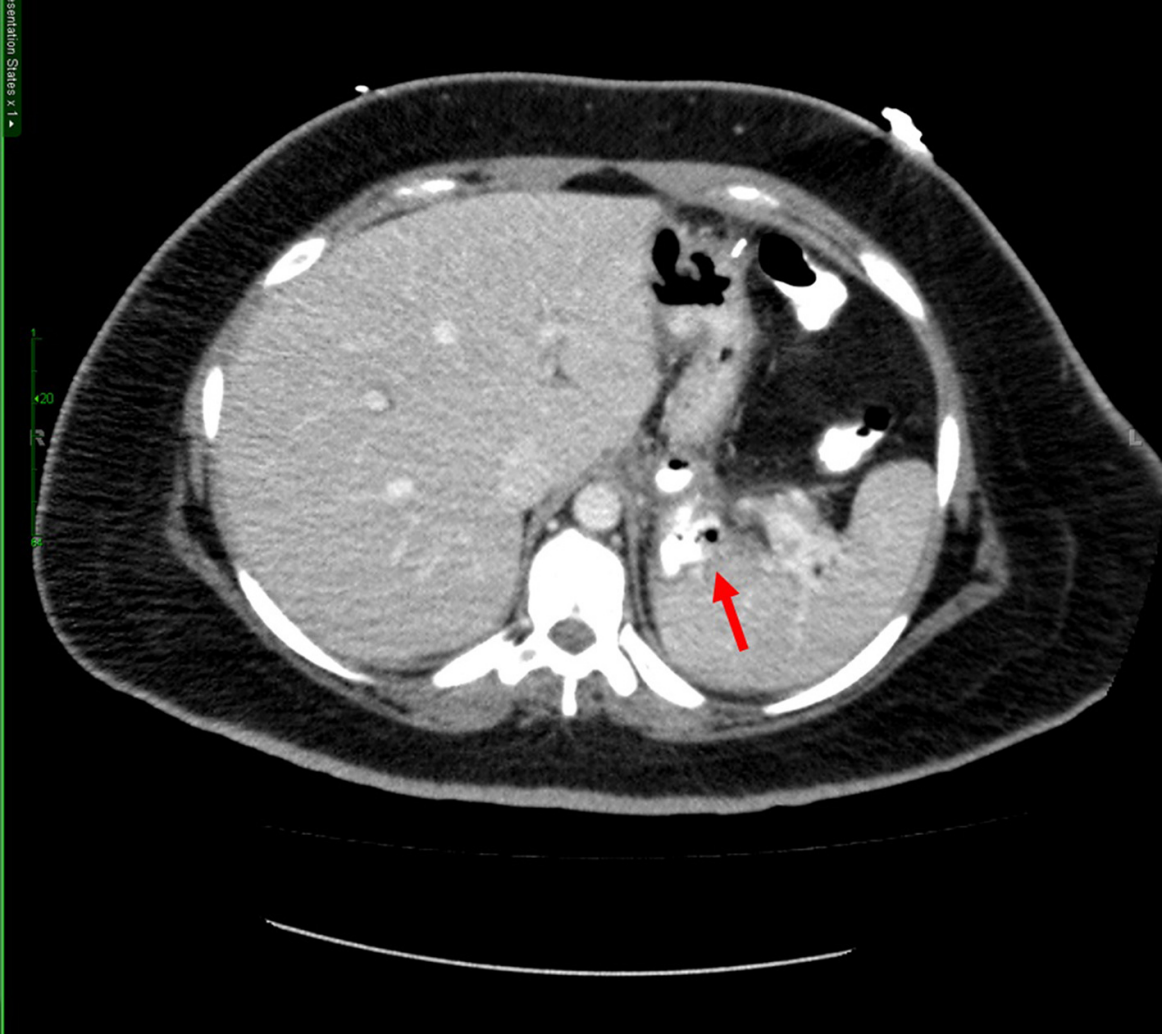
CT scan with contrast confirming leak, late phase. Arrow indicates extravasated extraluminal contrast and air representing leak on CT.

On post-operative day 12, the patient was readmitted to an outside tertiary care hospital for lightheadedness and shortness of breath and was found to have leukocytosis, with white blood cell count of 18,000 cells μl^–1^. The work-up included a CT scan with intravenous contrast of the chest, abdomen and pelvis, and the patient was diagnosed with a pulmonary embolism. The patient was immediately transferred to our centre for definitive care. When the patient arrived at our centre, the CT films from the outside hospital were reviewed by our radiologists and there was concern that there was air and a faint suggestion of oral contrast outside of the suture line (Figure 1). Given this finding, an UGI evaluation was ordered. During the early phase, no leak was observed, owing, in part, to the slow passage of 30 ml oral non-ionic contrast (Figure 2a). Some residual contrast from the outside hospital CT was present in the transverse and descending colon. Only after delayed imaging and with administration of additional non-ionic contrast for a total of about 65 ml (approximately 2 h after the start of the fluoroscopic examination) was there a faint suggestion of extravasated contrast, best seen below the left hemidiaphragm (Figure 2b). Follow-up CT scan with oral contrast confirmed the obvious leak (Figure 3).

The patient was treated definitively with endoscopic stent placement and clipping using an Ovesco clip (Ovesco Endoscopy AG, Tubingen, Germany) to close the leak. After an extended hospital course, she was discharged and is presently doing well.

## Discussion

Early detection of anastomotic and/or staple line leaks can lead to significant reductions in morbidity and mortality following bariatric surgery. Accurate and timely detection is vital to the patient’s outcome. Presently, there remains no universal diagnostic algorithm to assess for leak and use of imaging modalities can vary widely among clinicians. In a retrospective study and case series of 619 patients, of whom 20 had experienced a leak, CT with intravenous contrast had a sensitivity of 95% [95% confidence interval (CI) (82–99%)] and specificity of 100% [95% CI (91–100%)], while UGI had an inferior sensitivity of 79% [95% CI (62–90%)] and specificity of 95% [95% CI (85–99%)].^4^ Other, smaller studies comparing CT with fluoroscopy, however, have had mixed results regarding which diagnostic test is more sensitive.^5^ Our case report demonstrates that, despite appropriate contrast administration, it was not possible to adequately detect a leak by UGI examination alone. UGI examination followed immediately by CT imaging proved to be diagnostic in detecting this subtle finding.

UGI and CT both confer specific advantages. For example, a CT scan can identify other complications, such as abscesses and obstructions. Also, a CT scan is notably less dependent on patient and technologist compliance (respiratory motion, artefacts and positioning difficulties) and does not result in suboptimal examinations and subsequent false negatives, as in the case of UGI. Lateral and/or steep oblique positioning on the fluoroscopy table is not easy to accomplish in an immediate post-operative patient. A previous study of 43 oesophagectomy patients found that 83% tolerated CT scan better than fluoroscopy.^6^ In the future, image post-processing tools may assist with more accurate diagnosis on CT scan.^7,8^

It is important to note that the clinical situation should guide the decision to undergo imaging to find a leak. In particular, routine UGI examination in non-symptomatic patients has been found to have low sensitivity.^8-11^ UGI examination, on the other hand, may help define the size and location of the leak with a greater degree of precision, but typically cannot be performed until the following day if the patient becomes symptomatic at night owing to staffing limitations at many hospitals. We propose that CT evaluation, used in combination with UGI, may increase the overall sensitivity of detecting a leak, thereby improving patient outcomes and decreasing hospital utilization. Further studies are needed to evaluate and validate the use of combination modalities such as CT and UGI to better detect leaks in a bariatric surgery patient.

## Learning points

1. In a retrospective case series, CT scan has been found to have superior sensitivity and specificity to UGI evaluation for detecting a leak following bariatric surgery.
2. This case report demonstrates that CT evaluation can increase the sensitivity when combined with UGI.
3. Clinical suspicion and an understanding of the advantages and disadvantages of each diagnostic test should guide the diagnosis of a possible post-operative leak.

## Consent

Written informed consent for the case to be published (including images, case history and data) was obtained from the patient(s) for publication of this case report.

## References

[1] Fernandez AZ, DeMaria EJ, Tichansky DS, Kellum JM, Wolfe LG, Meador J, et al Experience with over 3,000 open and laparoscopic bariatric procedures: multivariate analysis of factors related to leak and resultant mortality. Surg Endosc 2004; 18: 193–7. 14691697 10.1007/s00464-003-8926-y

[2] Carucci LR, Turner MA, Conklin RC, DeMaria EJ, Kellum JM, Sugerman HJ, et al Roux-en-Y gastric bypass surgery for morbid obesity: evaluation of postoperative extraluminal leaks with upper gastrointestinal series. Radiology 2006; 238: 119–27. 16373763 10.1148/radiol.2381041557

[3] Bingham J, Shawhan R, Parker R, Wigboldy J, Sohn V Computed tomography scan versus upper gastrointestinal fluoroscopy for diagnosis of staple line leak following bariatric surgery. Am J Surg 2015; 209: 810–14. 25747433 10.1016/j.amjsurg.2015.01.004

[4] Sakran N, Goitein D, Raziel A, Keidar A, Beglaibter N, Grinbaum R, et al Gastric leaks after sleeve gastrectomy: a multicenter experience with 2,834 patients. Surg Endosc 2013; 27: 240–5. 22752283 10.1007/s00464-012-2426-x

[5] Upponi S, Ganeshan A, D’Costa H, Betts M, Maynard N, Bungay H, et al Radiological detection of post-oesophagectomy anastomotic leak—a comparison between multidetector CT and fluoroscopy. Br J Radiol 2008; 81: 545–8. 18559902 10.1259/bjr/30515892

[6] Picone D, Rusignuolo R, Midiri F, Lo Casto A, Vernuccio F, Pinto F, et al Imaging assessment of gastroduodenal perforations. Semin Ultrasound CT MR 2016; 37: 16–22. 26827734 10.1053/j.sult.2015.10.006

[7] Patlas MN, Alabousi A, Scaglione M, Romano L, Soto JA Cross-sectional imaging of nontraumatic peritoneal and mesenteric emergencies. Can Assoc Radiol J 2013; 64: 148–53. 23528385 10.1016/j.carj.2013.02.001

[8] Schiesser M, Guber J, Wildi S, Guber I, Weber M, Muller MK Utility of routine versus selective upper gastrointestinal series to detect anastomotic leaks after laparoscopic gastric bypass. Obes Surg 2011; 21: 1238–42. 20872254 10.1007/s11695-010-0284-y

[9] Bertelson NL, Myers JA Routine postoperative upper gastrointestinal fluoroscopy is unnecessary after laparoscopic adjustable gastric band placement. Surg Endosc 2010; 24: 2188–91. 20349088 10.1007/s00464-010-0924-2

[10] Kolakowski S, Kirkland ML, Schuricht AL Routine postoperative upper gastrointestinal series after Roux-en-Y gastric bypass: determination of whether it is necessary. Arch Surg 2007; 142: 930–4. 17938304 10.1001/archsurg.142.10.930

[11] Kim TH, Kim JH, Shin CI, Kim SH, Han JK, Choi BI CT findings suggesting anastomotic leak and predicting the recovery period following gastric surgery. Eur Radiol 2015; 25: 1958–66. 25708962 10.1007/s00330-015-3608-4

